# Follow-Up (Measurement) of Corrected QT Interval in Adult Patients before and after Lung Transplantation

**DOI:** 10.1155/2017/4519796

**Published:** 2017-11-06

**Authors:** Dirk Bandorski, Reinhard Hoeltgen, Nicole Becker, Winfried Padberg, Harilaos Bogossian, Christoph Wiedenroth, Matthias Arlt, Christian Hamm, Werner Seeger, Hossein Ardeschir Ghofrani, Matthias Hecker, Henning Gall, Konstantin Mayer

**Affiliations:** ^1^University of Giessen and Marburg Lung Center (UGMLC), The German Center for Lung Research (DZL), Klinikstrasse 33, 35392 Giessen, Germany; ^2^Klinikum Westmünsterland, St. Agnes-Hospital Bocholt Rhede, Medical Clinic 1-Cardiology/Electrophysiology, Barloer Weg 125, 46397 Bocholt, Germany; ^3^Department of General and Thoracic Surgery, University Hospital of Giessen, Rudolf-Buchheim-Strasse 7, 35392 Giessen, Germany; ^4^Klinikum Lüdenscheid, Universität Witten-Herdecke, Medizinische Klinik III, Paulmannhöherstr. 14, 58515 Lüdenscheid, Germany; ^5^Department of Thoracic Surgery, Kerckhoff Heart and Lung Center, Benekestrasse 2-8, 61231 Bad Nauheim, Germany; ^6^Department of Intensive Care Medicine, Kerckhoff Heart and Lung Center, Benekestrasse 2-8, 61231 Bad Nauheim, Germany; ^7^Medical Clinic 1, University Hospital Giessen and Marburg GmbH, Klinikstrasse 33, 35392 Giessen, Germany

## Abstract

**Background:**

Prolongation of the corrected QT (QTc) interval is well known for many drugs, some of which are an integral part of the therapeutic regimen after lung transplantation (LTX). Therefore, we investigated the QTc interval after LTX in the present study.

**Patients and Methods:**

The medical records of patients after LTX were studied for demographic data, indication of LTX, medication, and baseline and follow-up ECGs. The QT interval was corrected for the patient's heart rate using the different formulae of Bazett, Fridericia, Hodges, and Framingham.

**Results:**

Fifty-nine patients were included. The mean age ± SD was 55.6 ± 7.8 years (median 58 years). After LTX, QTc intervals showed no (relevant) changes during follow-up, even though all patients were treated with drugs (in combination) known to bear a risk of prolonged QTc interval and cortisone. The longest QTc intervals were obtained using Bazett's formula.

**Conclusion:**

The QTc interval did not increase under immunosuppressive medication after LTX in our cohort of patients. We speculate that the concurrent use of cortisone may shorten the QT(c) intervals or cancel out drug-induced prolongation of the QTc interval.

## 1. Introduction

Prolongation of the corrected QT interval (QTc) is well known for many drugs [1–4]. When checking CredibleMeds.org [5], an independent peer-reviewed online resource, for drug side effects, the macrolides azithromycin and clarithromycin, both of which are currently prescribed to regular patients with lung transplantation in our center, are listed within the drugs with a known risk to prolong QTc. Drugs are classified corresponding to their “Current TdP risk category” (“drugs with conditional TdP risk”: itraconazole, posaconazole, voriconazole, pantoprazole, and omeprazole; “drugs with possible TdP risk”: tacrolimus; and “drugs with known TdP risk”: azithromycin and clarithromycin). Additionally “Conditions for TdP” (“reduces elimination of a QT drug”: itraconazole, posaconazole, and voriconazole; “use with concomitant QT drug, use causes low serum Mg or K”: pantoprazole, omeprazole) are mentioned [5].

While the risk of drug-induced prolonged QT and the incidence of possible lethal Torsade de Pointes (TdP) arrhythmias are generally low with an annually reported rate of 0.8 to 1.2 per million persons years (reviewed in [6]), a recent study from Germany found the reported rate to be increasing from 0.26 to 2.5 and 4.0 per million per year in males and females, respectively, due to active surveillance [7]. Even so, in high risk populations such as lung-transplanted patients, due to exposure to many known drugs bearing the risk of prolonged QTc interval, the incidence may be dramatically higher [6]. A case report described witnessed TdP after the repeated administration of tacrolimus in an intensive care setting after transplantation [8].

Currently, the situation after transplantation is far from clear. In patients receiving a liver transplantation, the prevalence of prolonged QTc seems to be high before transplantation. An early study found prolonged QTc in 44 out of 53 patients with an improvement in 32 of the 44 patients after transplantation [9]. In a larger study of 269 patients with end-stage liver disease, the QTc was 449 ms but shortened after transplantation to 417 ms [10]. In a cohort of 249 patients before liver transplantation, 105 patients exhibited a prolonged QTc which normalized in 91 and shortened in further 14 patients after transplantation [11]. The cause of the prolongation of QTc and recovery after liver transplantation remained unclear, as MELD score, age, and sex could not predict prolonged QTc [10], and two authors speculated about an improvement of autonomic function [9, 10]. In contrast to the shortening of QTc in liver transplantation, in a cohort of 995 patients with stem cell transplantation, QTc increased from 426 ms to 441 ms after transplantation [12]. The authors identified an “at risk population” of pretransplant prolonged QTc, which exhibited a trend of posttransplant nonrelapse mortality [12]. There are contradicting reports in renal transplantation, reporting a shortening of QTc in 34 patients two weeks after transplantation [13] or prolongation of QTc in a cohort of 98 patients after a longer follow-up [14]. Therefore, the aim of our study was to elucidate the range of QTc interval in patients with lung transplantation under respective common medications.

## 2. Methods

We carried out a retrospective analysis of all cases of lung transplantation (LTX) at our institution between 1st August 1999 and 1st February 2013. Medical records were studied for demographic data, indication of LTX, medication, and baseline and follow-up ECGs. The study and the study design were approved by the Institutional Review Board (Ethikkommision des Universitätsklinikums Giessen und Marburg, reference number: 138/12). All patients were followed in our outpatient clinic after LTX.

### 2.1. Electrocardiogram

Twelve-lead ECGs with patients in supine position were performed by trained technicians and reviewed by two physicians (internist and cardiologist, NB and DB). Commercially available ECG devices (Model Schiller AT-10 plus; Schiller AG, Baar; Switzerland) were used for ECG recordings (paper speed: 50 mm/s; sensitivity of 10 mm/mV). The latest available pretransplant ECG and the latest ECGs within one and two years after LTX were analyzed with standard ECG nomenclature and definitions [15, 16]. ECGs were compared related to heart rhythm and the duration of QTc intervals.

The QT interval was measured from the earliest onset of QRS complex to the point of the T-wave offset, defined by a return of the terminal T-wave to the isoelectric TP baseline. In the presence of a U wave interrupting the T-wave, the terminal portion of the visible T-wave was extrapolated to the TP baseline to define the point of the T-wave offset. Each QT interval was corrected (QTc interval) for the patient's heart rate using the different established formulae of Bazett (QT/(RR)1/2) [17], Fridericia (QT/(RR)1/3) [18], Hodges (QT + 1.75 × (heart rate − 60)) [19], and Framingham (QT + 01.54 × (1 − RR)) [20]. The AHA/ACCF/HRS recommend measuring the QT interval in leads showing the longest QT, usually V2 or V3 [21]. The QT interval was assessed in these leads in our study.

Due to the different follow-up periods, a different number of follow-up ECGs per patient were available. The average number of ECGs per patient was six.

### 2.2. Statistics

Data are expressed as mean +/− standard deviation or median with the interquartile range (IQR) for normally or nonnormally distributed parameters, as appropriate. Paired sample *t*-tests were applied for changes in QTc interval over time. Spearman correlation analysis was performed. Statistical analysis was performed using SPSS 21.0 software (IBM Corp., Armonk, NY).

## 3. Results

### 3.1. Patients

Fifty-nine patients were included in the analyses. The mean age was 55.6 ± 7.8 years. The indications for LTX were cystic fibrosis in two patients, chronic obstructive lung disease (COPD) in 24 patients, lung fibrosis in 31 patients, lymphangioleiomyomatosis of the lung in one patient, and pulmonary hypertension in one patient. Twelve patients died during follow-up. Of these, three suffered from pneumonia with sepsis, two patients from respiratory insufficiency due to chronic transplant rejection, three patients from sepsis (abdominal, pancreatitis), two patients from malignoma, one patient from intracerebral hemorrhage, and one patient died from sudden cardiac death.

The mean time of follow-up in all patients was 28.7 ± 24.9 months. All patients were under medication with tacrolimus, mycophenolate mofetil (MMF), itraconazole, macrolides (azithromycin, clarithromycin), and prednisolone. Perioperatively and postoperatively, patients were treated with cortisone according to the following protocol: 500 mg methylprednisolone at the beginning of LTX (skin incision), 500 mg methylprednisolone before reperfusion of each lung, 3 × 125 mg methylprednisolone at the first day after LTX (8, 16, and 24 hours after reperfusion), prednisolone 1 mg/kg body weight at the second day after LTX with a reduction of 5 mg/day until 0.5 mg/kg body was reached up to the third month after LTX, 0.3 mg/kg body weight up to the sixth month after LTX, and the subsequent 0.2 mg/kg body weight. Four patients were on beta blocker medication.

### 3.2. Electrocardiogram

Depending on the formula used, in all patients (*n* = 59) the QTc interval before LTX was between 400 and 425 msec (Table 1).

Patients (*n* = 44) with an ECG before and within one year (3–12 months) after LTX showed similar results for the QTc interval. QTc interval did not change within one year (Table 2).

In 22 patients, the QTc interval two years after LTX (in comparison with QTc interval before LTX) was near-constant: Bazett: 415 ± 40 msec versus 398 ± 40 msec (*p* = 0.018), Fridericia 393 ± 38 msec versus 383 ± 38 msec (*p* = 0.159), Hodges 396 ± 35 msec versus 385 ± 36 msec (*p* = 0.094), and Framingham 394 ± 36 msec versus 385 ± 35 msec (*p* = 0.145). Heart rate was 84 ± 16 beats/min before and 79 ± 17 beats/min after LTX (*p* = 0.199). The longest QTc intervals were obtained by calculation with Bazett's formula.

Serum levels of tacrolimus and duration of the QTc interval for the first five ECGs were correlated in all patients. The association between serum levels of tacrolimus (in mean 10 ± 5 *μ*g/l) was low, with a correlation coefficient between 0.03 and 0.2, without statistical significance.

## 4. Discussion

To the best of our knowledge, this is the first study to evaluate long-term changes of the QTc interval in patients after LTX. Normal values of the QTc interval are 460 ms in women and 450 ms in men [21]. In our patients, the QTc interval appeared to be within the normal range before and after LTX. In contrast to our expectations, QTc interval was not prolonged in our cohort, despite all patients on multiple medications being known to bear a risk of prolonged QTc.

The effect of transplantation on QTc interval is not homogenous. Patients with end-stage liver disease were found to exhibit a prolonged QTc interval which shortened after liver transplantation. It is unclear why the QTc was prolonged before transplantation and what the drivers of the shortening effect of transplantation were [9–11]. The QTc interval was reported to shorten shortly after kidney transplantation but another group found an increase in QTc interval after a longer term follow-up [13, 14]. Again, the drivers of the changes in QTc interval remain open to speculation. However, in a large cohort of patients with stem cell transplantation, QTc interval increased by 16 msec to an average of 441 ms [12]. Patients with a prolonged QTc interval before transplant exhibited a risk of nontransplant associated complications after engraftment [12]. Interestingly, the study by Babuty et al. showed progressive shortening of the QTc interval during follow-up after cardiac transplantation. We caution comparing their results to those of other groups of transplanted patients, as the transplanted heart is denervated during surgical transplantation, leading to reduced suppression of the autonomic nervous system and QTc interval shortening [22].

In our study, the QTc interval was evaluated using the formulae of Bazett, Fridericia, Hodges, and Framingham. The use of different formulae to estimate QTc duration was analyzed in the studies of Luo et al. [23–25]. In accordance with their studies, for our patients, Bazett's formula revealed the longest QTc interval and overestimated the QTc interval relative to the other formulae (Fridericia, Hodges, and Framingham) [23–25]. Based on their results, Luo et al. generally recommend the use of Fridericia's formula for HR < 60 bpm and Hodges's for all other HR [23]. Chiladakis et al. concluded that Hodges's formula seems most appropriate when assessing the QTc interval and recommended avoiding Bazett's formula for the assessment of QTc intervals at heart rates far from 60 beats/min [24]. The recommendation of Luo et al. seems practicable for daily routine.

Interestingly, the QTc interval in our patients was not prolonged during follow-up, even though all patients were treated with drugs (in combination) known to bear a risk of prolonging QTc interval and cortisone. A review of the literature led us to propose the theory that cortisone seems to shorten the QTc interval or cancel out drug-induced prolongation of the QTc interval as at least one basic science [26] and one clinical paper [27] support this assumption. Based on this observation we performed a prospective study to analyze the influence of cortisone on the QTc interval (ClinicalTrials.gov ID: NCT03082339, Protocol ID: QTc25416). We investigate multiple sclerosis patients without a history of cardiovascular diseases. Actually 5 patients are included. The QTc interval shortened 27 msec under medication with cortisone and prolonged 11 msec one day after discontinuation of the medication.

The common pathophysiological basis between congenital long QT syndrome (cLQTS) and acquired long QT syndrome (aLQTS, Typ 2) is the HERG gene (human ether-a-go-go related gene). HERG is responsible for the expression of hERG channels (IKr) that are operative for the repolarization [28, 29]. Mutation of the HERG gene leads to cLQTS, and a drug-induced block of the channel causes aLQTS.

Peal et al. analyzed the chemical suppression of long QT syndrome (Type 2) using an* in vivo* zebrafish model [26]. Their study revealed that flurandrenolide reproducibly suppressed the long QT phenotype via the glucocorticoid signaling pathway. In contrast to treatment with dexamethasone and testosterone, treatment with pure mineralocorticoid deoxycorticosterone acetate did not suppress the long QT phenotype. Knockdown of the glucocorticoid receptor or, conversely, of the androgen receptor showed that flurandrenolide acting through the glucocorticoid receptor shortens ventricular action potentials. The mechanism is distinct from trafficking rescue of the defective zebrafish-ERG channel. The authors suggested that a drug normalizing repolarization would be a novel therapeutic tool in long QT syndrome and concluded that glucocorticoids could be expected to aid in the acute management of patients with long QT syndrome, for example, in episodes of arrhythmic storm. In addition, corticoid-induced normalization of the QT interval is reported in a patient with drug-induced prolongation of the QTc interval [27]. Brostoff and Lockwood reported a patient suffering from mucocutaneous leishmaniasis treated with sodium stibogluconate [27]. During therapy, the QTc interval was prolonged and returned to normal within 4 days after starting glucocorticoid therapy with prednisolone 20 mg twice daily.

The results of the basic science study and the clinical report support our hypothesis of shortening the QTc interval or canceling out drug-induced prolongation of the QTc interval with cortisone.

The limitations of the current study consist of the moderate sample size (*n* = 59). Basically, our study was not designed to determine the molecular substrates or investigate the mechanisms of cortisone on QTc interval. It should be remarked that the cortisone dose from the second year after LTX lies below the Cushing threshold dose. Different effects must be taken into consideration.

## 5. Conclusion

The QTc interval does not increase under immunosuppressive medication after LTX. The authors admit that the number of patients is too low to make an authoritative statement. Cortisone seems to shorten the QT(c) interval or cancel out drug-induced prolongation of the QTc interval.

## Figures and Tables

**Table 1:** QTc interval before lung transplantation (LTX) in all patients. Data are expressed as mean +/− standard deviation.

ECG before LTX (*n* = 59)				
Bazett	Fridericia	Hodges	Framingham	Heart rate
425 ± 46 msec	400 ± 42 msec	403 ± 37 msec	401 ± 36 msec	87 ± 16 beats/min

**Table 2:** QTc interval within one year after lung transplantation (LTX). Data are expressed as mean +/− standard deviation.

ECG before LTX (*n* = 44)				
Bazett	Fridericia	Hodges	Framingham	Heart rate
422 ± 47 msec	397 ± 42 msec	401 ± 37 msec	397 ± 36 msec	87 ± 18 beats/min

ECG within one year after LTX (*n* = 44)				
Bazett	Fridericia	Hodges	Framingham	Heart rate

418 ± 41 msec	390 ± 38 msec	400 ± 33 msec	388 ± 30 msec	93 ± 24 beats/min
*p* = 0.589	*p* = 0.303	*p* = 0.859	*p* = 0.152	*p* = 0.102
